# 2-*p*-Tolyl-1-*p*-tolyl­methyl-1*H*-benzimidazole

**DOI:** 10.1107/S1600536809021175

**Published:** 2009-06-27

**Authors:** Ting Liu

**Affiliations:** a Biology and Chemistry Department, Nanchang University College of Science and Technology, Nanchang 330029, People’s Republic of China

## Abstract

The asymmetric unit of the title compound, C_22_H_20_N_2_, contains two crystallographically independent mol­ecules in which the planar benzimidazole ring systems are oriented with respect to the adjacent tolyl rings at dihedral angles of 47.08 (8)/76.85 (8) and 39.52 (9)/87.49 (9)°, while the dihedral angles between the tolyl rings are 73.99 (3) and 81.51 (9)°. In the crystal structure, pairs of inter­molecular C—H⋯N inter­actions link one of the asymmetric mol­ecules into centrosymmetric dimers through *R*_2_^2^(8) ring motifs.

## Related literature

For general background to the biological and pharmaceutical activities of benzimidazole derivatives, see: Matsuno *et al.* (2000 ▶). Garuti *et al.* (1999 ▶). For related structures, see: Tlahuext *et al.* (2007 ▶); Chen & Ruan (2007 ▶). For bond-length data, see: Allen *et al.* (1987 ▶). For ring-motifs, see: Bernstein *et al.* (1995 ▶).
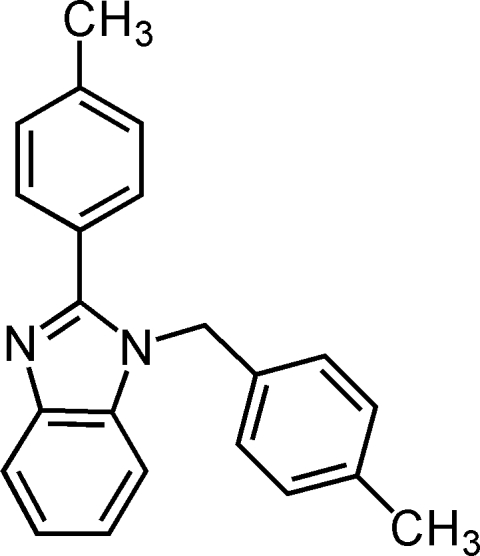


## Experimental

### 

#### Crystal data

C_22_H_20_N_2_
*M* *_r_* = 312.40
Triclinic, 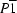

*a* = 9.7046 (19) Å
*b* = 10.457 (2) Å
*c* = 17.825 (4) Å
α = 84.12 (3)°
β = 81.44 (3)°
γ = 75.87 (3)°
*V* = 1730.4 (7) Å^3^
*Z* = 4
Mo *K*α radiation
μ = 0.07 mm^−1^
*T* = 294 K
0.20 × 0.18 × 0.15 mm

#### Data collection

Rigaku SCXmini diffractometer
Absorption correction: multi-scan (*CrystalClear*; Rigaku, 2005 ▶) *T*_min_ = 0.984, *T*_max_ = 0.988
16221 measured reflections
6773 independent reflections
3812 reflections with *I* > 2σ(*I*)
*R*_int_ = 0.057

#### Refinement

*R*[*F* ^2^ > 2σ(*F* ^2^)] = 0.065
*wR*(*F* ^2^) = 0.173
*S* = 1.01
6773 reflections
434 parameters
H-atom parameters constrained
Δρ_max_ = 0.23 e Å^−3^
Δρ_min_ = −0.16 e Å^−3^

### 

Data collection: *CrystalClear* (Rigaku, 2005 ▶); cell refinement: *CrystalClear*; data reduction: *CrystalClear*; program(s) used to solve structure: *SHELXS97* (Sheldrick, 2008 ▶); program(s) used to refine structure: *SHELXL97* (Sheldrick, 2008 ▶); molecular graphics: *SHELXTL* (Sheldrick, 2008 ▶); software used to prepare material for publication: *SHELXL97*.

## Supplementary Material

Crystal structure: contains datablocks I, global. DOI: 10.1107/S1600536809021175/hk2704sup1.cif

Structure factors: contains datablocks I. DOI: 10.1107/S1600536809021175/hk2704Isup2.hkl

Additional supplementary materials:  crystallographic information; 3D view; checkCIF report

## Figures and Tables

**Table 1:** Hydrogen-bond geometry (Å, °)

*D*—H⋯*A*	*D*—H	H⋯*A*	*D*⋯*A*	*D*—H⋯*A*
C35—H35*A*⋯N3^i^	0.93	2.60	3.491 (3)	162

Symmetry code: (i) 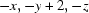.

**Fig. 1.**
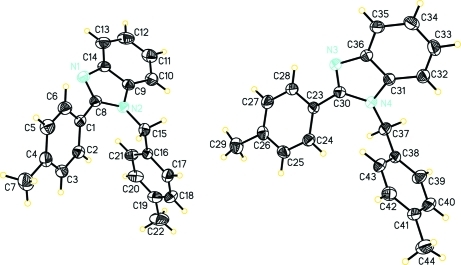
The molecular structure of the title molecule, with the atom-numbering scheme. Displacement ellipsoids are drawn at the 30% probability level.

**Fig. 2.**
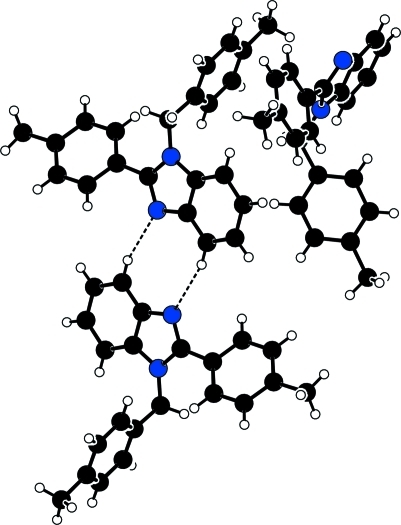
A partial packing diagram of the title compound. Hydrogen bonds are shown as dashed lines.

## supplementary crystallographic information

### Comment

Benzimidazole derivatives have attracted considerable attention because of their biological and pharmaceutical activities (Matsuno *et al.*,  2000; Garuti *et al.*,  1999). In addition, they play an important role in the development of coordination chemistry. Many derivatives of benzimidazole have been prepared and their complexes have been studied (Tlahuext *et al.*,  2007; Chen & Ruan, 2007). We report herein the crystal structure of the title compound.

The asymmetric unit of the title compound contains two crystallographically independent molecules (Fig. 1), in which the bond lengths (Allen *et al.*, 1987) and angles are within normal ranges. Rings A (C1-C6), B (N1/N2/C8/C9/C14), C (C9-C14), D (C16-C21) and E (C23-C28), F (N3/N4/C30/C31/C36), G (C31-C36), H (C38-C43) are, of course, planar. The planar benzimidazole ring systems, (N1/N2/C8-C14) and (N3/N4/C30-C36),  are oriented with respect to the adjacent rings, A, D and E, H, at dihedral angles of 47.08 (8), 76.85 (8) ° and 39.52 (9), 87.49 (9) °, respectively, while the dihedral angles between rings A, D and E, H are 73.99 (3) and 81.51 (9) °, respectively.

In the crystal structure, intermolecular C-H···N interactions (Table 1) link the molecules into centrosymmetric dimers through *R*_2_^2^(8) ring motifs (Bernstein *et al.*, 1995) (Fig. 2), in which they may be effective in the stabilization of the structure.

### Experimental

For the preparation of the title compound, 4-methylbenzaldehyde(0.96 g, 8 mmol) was added to a solution of *o*-phenylenediamine (0.432 g, 4 mmol) in ethanol (20 ml). The mixture was refluxed with stirring for 4 h. When cooled to room temperature, the resultant solution was filtered and allowed to evaporate slowly. Crystals suitable for X-ray analysis were obtained after several weeks.

### Refinement

H atoms were positioned geometrically, with C-H = 0.93, 0.97 and 0.96 Å for aromatic, methylene and methyl H, respectively, and constrained to ride on their parent atoms, with U_iso_(H) = 1.2U_eq_(C).

### Crystal data

**Table:**

C_22_H_20_N_2_	*Z* = 4
*M**_r_* = 312.40	*F*(000) = 664
Triclinic, *P*1	*D*_x_ = 1.199 Mg m^−^^3^
Hall symbol: -P 1	Mo *K*α radiation, λ = 0.71073 Å
*a* = 9.7046 (19) Å	Cell parameters from 2562 reflections
*b* = 10.457 (2) Å	θ = 3.0–27.5°
*c* = 17.825 (4) Å	µ = 0.07 mm^−^^1^
α = 84.12 (3)°	*T* = 294 K
β = 81.44 (3)°	Prism, yellow
γ = 75.87 (3)°	0.20 × 0.18 × 0.15 mm
*V* = 1730.4 (7) Å^3^	

### Data collection

**Table:**

Rigaku SCXmini diffractometer	6773 independent reflections
Radiation source: fine-focus sealed tube	3812 reflections with *I* > 2σ(*I*)
graphite	*R*_int_ = 0.057
Detector resolution: 13.6612 pixels mm^-1^	θ_max_ = 26.0°, θ_min_ = 3.0°
ω scans	*h* = −11→11
Absorption correction: multi-scan (*CrystalClear*; Rigaku, 2005)	*k* = −12→12
*T*_min_ = 0.984, *T*_max_ = 0.988	*l* = −21→21
16221 measured reflections	

### Refinement

**Table:**

Refinement on *F*^2^	Secondary atom site location: difference Fourier map
Least-squares matrix: full	Hydrogen site location: inferred from neighbouring sites
*R*[*F*^2^ > 2σ(*F*^2^)] = 0.065	H-atom parameters constrained
*wR*(*F*^2^) = 0.173	*w* = 1/[σ^2^(*F*_o_^2^) + (0.0746*P*)^2^ + 0.1986*P*] where *P* = (*F*_o_^2^ + 2*F*_c_^2^)/3
*S* = 1.01	(Δ/σ)_max_ = 0.001
6773 reflections	Δρ_max_ = 0.23 e Å^−^^3^
434 parameters	Δρ_min_ = −0.16 e Å^−^^3^
0 restraints	Extinction correction: *SHELXL97* (Sheldrick, 2008), Fc^*^=kFc[1+0.001xFc^2^λ^3^/sin(2θ)]^-1/4^
Primary atom site location: structure-invariant direct methods	Extinction coefficient: 0.0175 (19)

### Special details

**Table:**

Geometry. All e.s.d.'s (except the e.s.d. in the dihedral angle between two l.s. planes) are estimated using the full covariance matrix. The cell e.s.d.'s are taken into account individually in the estimation of e.s.d.'s in distances, angles and torsion angles; correlations between e.s.d.'s in cell parameters are only used when they are defined by crystal symmetry. An approximate (isotropic) treatment of cell e.s.d.'s is used for estimating e.s.d.'s involving l.s. planes.
Refinement. Refinement of *F*^2^ against ALL reflections. The weighted *R*-factor *wR* and goodness of fit *S* are based on *F*^2^, conventional *R*-factors *R* are based on *F*, with *F* set to zero for negative *F*^2^. The threshold expression of *F*^2^ > σ(*F*^2^) is used only for calculating *R*-factors(gt) *etc*. and is not relevant to the choice of reflections for refinement. *R*-factors based on *F*^2^ are statistically about twice as large as those based on *F*, and *R*- factors based on ALL data will be even larger.

### Fractional atomic coordinates and isotropic or equivalent isotropic displacement parameters (Å^2^)

**Table:**

	*x*	*y*	*z*	*U*_iso_*/*U*_eq_	
N1	0.6191 (2)	1.6298 (2)	−0.44528 (12)	0.0553 (6)	
N2	0.6780 (2)	1.57278 (19)	−0.32714 (11)	0.0453 (5)	
N3	0.1316 (2)	1.0170 (2)	0.07446 (11)	0.0479 (5)	
N4	0.2184 (2)	0.98105 (19)	0.18661 (11)	0.0424 (5)	
C1	0.8163 (3)	1.4336 (2)	−0.43273 (14)	0.0464 (6)	
C2	0.9553 (3)	1.4080 (3)	−0.41494 (14)	0.0542 (7)	
H2A	0.9774	1.4587	−0.3806	0.065*	
C3	1.0614 (3)	1.3078 (3)	−0.44762 (15)	0.0586 (8)	
H3B	1.1543	1.2925	−0.4355	0.070*	
C4	1.0298 (3)	1.2301 (3)	−0.49837 (16)	0.0588 (8)	
C5	0.8919 (3)	1.2560 (3)	−0.51599 (16)	0.0650 (8)	
H5A	0.8699	1.2052	−0.5504	0.078*	
C6	0.7857 (3)	1.3558 (3)	−0.48363 (15)	0.0576 (7)	
H6A	0.6930	1.3710	−0.4960	0.069*	
C7	1.1466 (3)	1.1202 (3)	−0.53379 (19)	0.0848 (10)	
H7A	1.2352	1.1176	−0.5152	0.127*	
H7B	1.1578	1.1364	−0.5881	0.127*	
H7C	1.1206	1.0371	−0.5205	0.127*	
C8	0.7038 (3)	1.5452 (2)	−0.40265 (14)	0.0466 (6)	
C9	0.5663 (3)	1.6844 (3)	−0.32230 (15)	0.0479 (6)	
C10	0.4940 (3)	1.7545 (3)	−0.26059 (17)	0.0612 (8)	
H10A	0.5174	1.7295	−0.2116	0.073*	
C11	0.3857 (3)	1.8631 (3)	−0.2755 (2)	0.0767 (9)	
H11A	0.3343	1.9126	−0.2357	0.092*	
C12	0.3519 (3)	1.9000 (3)	−0.3491 (2)	0.0804 (10)	
H12A	0.2792	1.9744	−0.3575	0.096*	
C13	0.4237 (3)	1.8291 (3)	−0.40956 (19)	0.0700 (9)	
H13A	0.4002	1.8542	−0.4585	0.084*	
C14	0.5320 (3)	1.7190 (3)	−0.39567 (16)	0.0523 (7)	
C15	0.7380 (3)	1.4943 (3)	−0.26301 (14)	0.0507 (7)	
H15A	0.7855	1.4068	−0.2790	0.061*	
H15B	0.6599	1.4847	−0.2236	0.061*	
C16	0.8433 (2)	1.5486 (2)	−0.22885 (13)	0.0437 (6)	
C17	0.9021 (3)	1.4811 (3)	−0.16589 (15)	0.0618 (8)	
H17A	0.8757	1.4037	−0.1457	0.074*	
C18	0.9984 (3)	1.5259 (3)	−0.13277 (17)	0.0718 (9)	
H18A	1.0368	1.4775	−0.0909	0.086*	
C19	1.0397 (3)	1.6404 (3)	−0.15976 (19)	0.0659 (8)	
C20	0.9818 (3)	1.7078 (3)	−0.22217 (19)	0.0662 (8)	
H20A	1.0078	1.7857	−0.2417	0.079*	
C21	0.8854 (3)	1.6627 (3)	−0.25690 (16)	0.0554 (7)	
H21A	0.8489	1.7101	−0.2995	0.066*	
C22	1.1441 (4)	1.6895 (4)	−0.1216 (2)	0.1041 (13)	
H22A	1.1721	1.6294	−0.0791	0.156*	
H22B	1.0992	1.7755	−0.1041	0.156*	
H22C	1.2272	1.6948	−0.1573	0.156*	
C23	0.3362 (3)	1.1114 (2)	0.07900 (13)	0.0431 (6)	
C24	0.4795 (3)	1.0759 (3)	0.09004 (14)	0.0495 (7)	
H24A	0.5119	1.0032	0.1228	0.059*	
C25	0.5746 (3)	1.1477 (3)	0.05280 (14)	0.0541 (7)	
H25A	0.6702	1.1226	0.0611	0.065*	
C26	0.5304 (3)	1.2565 (3)	0.00336 (14)	0.0518 (7)	
C27	0.3868 (3)	1.2908 (3)	−0.00714 (15)	0.0581 (7)	
H27A	0.3543	1.3637	−0.0397	0.070*	
C28	0.2916 (3)	1.2198 (3)	0.02942 (15)	0.0532 (7)	
H28A	0.1962	1.2447	0.0208	0.064*	
C29	0.6345 (3)	1.3335 (3)	−0.03835 (17)	0.0734 (9)	
H29A	0.5847	1.4046	−0.0699	0.110*	
H29B	0.7084	1.2761	−0.0695	0.110*	
H29C	0.6768	1.3690	−0.0022	0.110*	
C30	0.2308 (2)	1.0356 (2)	0.11300 (14)	0.0438 (6)	
C31	0.1011 (2)	0.9250 (2)	0.19544 (14)	0.0423 (6)	
C32	0.0355 (3)	0.8616 (2)	0.25793 (15)	0.0526 (7)	
H32A	0.0700	0.8477	0.3048	0.063*	
C33	−0.0839 (3)	0.8205 (3)	0.24641 (17)	0.0615 (8)	
H33A	−0.1311	0.7774	0.2867	0.074*	
C34	−0.1361 (3)	0.8413 (3)	0.17649 (18)	0.0608 (8)	
H34A	−0.2171	0.8120	0.1714	0.073*	
C35	−0.0707 (3)	0.9041 (2)	0.11474 (16)	0.0538 (7)	
H35A	−0.1055	0.9172	0.0680	0.065*	
C36	0.0495 (3)	0.9472 (2)	0.12493 (14)	0.0447 (6)	
C37	0.2984 (3)	0.9895 (2)	0.24814 (13)	0.0471 (6)	
H37A	0.2310	1.0158	0.2929	0.056*	
H37B	0.3507	1.0582	0.2336	0.056*	
C38	0.4024 (2)	0.8630 (2)	0.26853 (13)	0.0428 (6)	
C39	0.4396 (3)	0.8375 (3)	0.34111 (15)	0.0651 (8)	
H39A	0.3976	0.8975	0.3777	0.078*	
C40	0.5393 (3)	0.7230 (3)	0.36022 (16)	0.0699 (9)	
H40A	0.5623	0.7076	0.4097	0.084*	
C41	0.6042 (3)	0.6326 (3)	0.30811 (16)	0.0539 (7)	
C42	0.5665 (3)	0.6578 (3)	0.23607 (16)	0.0546 (7)	
H42A	0.6086	0.5974	0.1997	0.065*	
C43	0.4675 (3)	0.7708 (2)	0.21636 (14)	0.0483 (6)	
H43A	0.4442	0.7851	0.1670	0.058*	
C44	0.7142 (3)	0.5098 (3)	0.3292 (2)	0.0820 (10)	
H44A	0.7274	0.5095	0.3815	0.123*	
H44B	0.8035	0.5090	0.2975	0.123*	
H44C	0.6816	0.4329	0.3219	0.123*	

### Atomic displacement parameters (Å^2^)

**Table:**

	*U*^11^	*U*^22^	*U*^33^	*U*^12^	*U*^13^	*U*^23^
N1	0.0517 (13)	0.0609 (14)	0.0511 (14)	−0.0031 (11)	−0.0162 (11)	−0.0040 (12)
N2	0.0463 (12)	0.0466 (12)	0.0433 (12)	−0.0079 (10)	−0.0098 (9)	−0.0052 (10)
N3	0.0442 (12)	0.0577 (13)	0.0432 (12)	−0.0158 (10)	−0.0102 (10)	0.0059 (11)
N4	0.0433 (12)	0.0450 (12)	0.0383 (12)	−0.0096 (10)	−0.0078 (9)	0.0019 (10)
C1	0.0471 (15)	0.0490 (15)	0.0415 (14)	−0.0080 (12)	−0.0083 (11)	0.0000 (12)
C2	0.0547 (17)	0.0587 (17)	0.0478 (16)	−0.0087 (14)	−0.0081 (13)	−0.0055 (14)
C3	0.0518 (17)	0.0625 (18)	0.0529 (17)	−0.0027 (14)	−0.0039 (13)	0.0059 (15)
C4	0.067 (2)	0.0468 (16)	0.0526 (17)	−0.0016 (14)	0.0037 (14)	0.0008 (14)
C5	0.074 (2)	0.0587 (18)	0.0642 (19)	−0.0117 (16)	−0.0110 (16)	−0.0164 (16)
C6	0.0547 (17)	0.0605 (18)	0.0587 (17)	−0.0118 (14)	−0.0110 (13)	−0.0079 (15)
C7	0.088 (2)	0.068 (2)	0.082 (2)	0.0055 (18)	0.0055 (19)	−0.0086 (18)
C8	0.0438 (15)	0.0521 (16)	0.0460 (15)	−0.0111 (13)	−0.0110 (12)	−0.0054 (13)
C9	0.0412 (14)	0.0506 (15)	0.0553 (17)	−0.0136 (12)	−0.0069 (12)	−0.0109 (13)
C10	0.0611 (18)	0.0663 (19)	0.0587 (18)	−0.0145 (16)	−0.0080 (14)	−0.0167 (16)
C11	0.062 (2)	0.075 (2)	0.090 (3)	−0.0025 (17)	−0.0051 (18)	−0.035 (2)
C12	0.058 (2)	0.072 (2)	0.106 (3)	0.0082 (16)	−0.0221 (19)	−0.023 (2)
C13	0.0563 (18)	0.070 (2)	0.079 (2)	0.0031 (16)	−0.0216 (16)	−0.0095 (18)
C14	0.0415 (15)	0.0579 (17)	0.0590 (17)	−0.0085 (13)	−0.0117 (13)	−0.0104 (14)
C15	0.0563 (17)	0.0509 (16)	0.0454 (15)	−0.0164 (13)	−0.0041 (13)	−0.0002 (13)
C16	0.0439 (15)	0.0468 (15)	0.0385 (14)	−0.0067 (12)	−0.0059 (11)	−0.0026 (12)
C17	0.0648 (19)	0.0699 (19)	0.0512 (17)	−0.0202 (16)	−0.0081 (14)	0.0060 (15)
C18	0.068 (2)	0.093 (2)	0.0534 (18)	−0.0091 (18)	−0.0245 (15)	−0.0008 (18)
C19	0.0518 (18)	0.069 (2)	0.079 (2)	−0.0039 (15)	−0.0170 (16)	−0.0234 (18)
C20	0.0556 (18)	0.0503 (17)	0.096 (2)	−0.0115 (14)	−0.0193 (17)	−0.0082 (17)
C21	0.0542 (17)	0.0454 (16)	0.0666 (19)	−0.0085 (13)	−0.0171 (14)	0.0017 (14)
C22	0.074 (2)	0.120 (3)	0.132 (3)	−0.016 (2)	−0.045 (2)	−0.043 (3)
C23	0.0431 (14)	0.0497 (15)	0.0377 (14)	−0.0132 (12)	−0.0056 (11)	−0.0029 (12)
C24	0.0519 (16)	0.0528 (16)	0.0457 (15)	−0.0136 (13)	−0.0119 (12)	−0.0001 (13)
C25	0.0463 (16)	0.0699 (19)	0.0513 (17)	−0.0203 (14)	−0.0062 (13)	−0.0114 (15)
C26	0.0608 (18)	0.0561 (17)	0.0439 (15)	−0.0262 (14)	0.0004 (13)	−0.0080 (14)
C27	0.0647 (19)	0.0566 (17)	0.0536 (17)	−0.0194 (15)	−0.0102 (14)	0.0100 (14)
C28	0.0463 (16)	0.0592 (17)	0.0540 (16)	−0.0144 (13)	−0.0101 (13)	0.0069 (14)
C29	0.083 (2)	0.083 (2)	0.067 (2)	−0.0471 (19)	−0.0051 (16)	−0.0010 (17)
C30	0.0393 (14)	0.0464 (15)	0.0436 (15)	−0.0065 (12)	−0.0057 (11)	−0.0020 (12)
C31	0.0412 (14)	0.0371 (13)	0.0452 (15)	−0.0057 (11)	−0.0039 (11)	0.0017 (12)
C32	0.0537 (17)	0.0460 (15)	0.0538 (17)	−0.0070 (13)	−0.0056 (13)	0.0035 (13)
C33	0.0564 (18)	0.0562 (17)	0.067 (2)	−0.0160 (14)	0.0065 (15)	0.0066 (15)
C34	0.0439 (16)	0.0567 (17)	0.081 (2)	−0.0146 (13)	−0.0078 (15)	0.0048 (16)
C35	0.0448 (16)	0.0552 (17)	0.0631 (18)	−0.0140 (13)	−0.0148 (13)	0.0045 (14)
C36	0.0419 (14)	0.0452 (15)	0.0446 (15)	−0.0061 (12)	−0.0080 (11)	0.0027 (12)
C37	0.0542 (16)	0.0481 (15)	0.0392 (14)	−0.0089 (13)	−0.0113 (12)	−0.0044 (12)
C38	0.0414 (14)	0.0524 (15)	0.0370 (14)	−0.0163 (12)	−0.0063 (11)	0.0002 (12)
C39	0.0653 (19)	0.083 (2)	0.0389 (16)	0.0035 (16)	−0.0135 (13)	−0.0090 (15)
C40	0.0641 (19)	0.097 (2)	0.0414 (16)	−0.0047 (18)	−0.0168 (14)	0.0069 (17)
C41	0.0436 (15)	0.0598 (18)	0.0582 (18)	−0.0130 (13)	−0.0142 (13)	0.0097 (15)
C42	0.0530 (17)	0.0521 (17)	0.0584 (18)	−0.0104 (13)	−0.0087 (13)	−0.0052 (14)
C43	0.0586 (17)	0.0500 (16)	0.0391 (14)	−0.0144 (13)	−0.0142 (12)	0.0000 (13)
C44	0.070 (2)	0.075 (2)	0.095 (3)	−0.0042 (17)	−0.0285 (18)	0.0143 (19)

### Geometric parameters (Å, °)

**Table:**

N1—C8	1.315 (3)	C21—C20	1.389 (4)
N1—C14	1.388 (3)	C21—H21A	0.9300
N2—C8	1.379 (3)	C22—H22A	0.9600
N2—C9	1.386 (3)	C22—H22B	0.9600
N2—C15	1.446 (3)	C22—H22C	0.9600
N3—C30	1.324 (3)	C23—C24	1.388 (3)
N3—C36	1.383 (3)	C23—C28	1.386 (3)
N4—C30	1.376 (3)	C23—C30	1.467 (3)
N4—C31	1.384 (3)	C24—C25	1.382 (3)
N4—C37	1.456 (3)	C24—H24A	0.9300
C1—C2	1.387 (3)	C25—H25A	0.9300
C1—C6	1.387 (3)	C26—C25	1.387 (4)
C2—C3	1.384 (4)	C26—C29	1.508 (4)
C2—H2A	0.9300	C27—C26	1.388 (4)
C3—H3B	0.9300	C27—H27A	0.9300
C4—C3	1.388 (4)	C28—C27	1.373 (3)
C4—C5	1.376 (4)	C28—H28A	0.9300
C4—C7	1.518 (4)	C29—H29A	0.9600
C5—H5A	0.9300	C29—H29B	0.9600
C6—C5	1.381 (4)	C29—H29C	0.9600
C6—H6A	0.9300	C31—C32	1.389 (3)
C7—H7A	0.9600	C31—C36	1.398 (3)
C7—H7B	0.9600	C32—C33	1.378 (4)
C7—H7C	0.9600	C32—H32A	0.9300
C8—C1	1.474 (3)	C33—H33A	0.9300
C9—C10	1.387 (4)	C34—C33	1.391 (4)
C9—C14	1.388 (4)	C34—C35	1.376 (4)
C10—C11	1.380 (4)	C34—H34A	0.9300
C10—H10A	0.9300	C35—H35A	0.9300
C11—H11A	0.9300	C36—C35	1.390 (3)
C12—C11	1.393 (4)	C37—C38	1.504 (3)
C12—H12A	0.9300	C37—H37A	0.9700
C13—C12	1.373 (4)	C37—H37B	0.9700
C13—H13A	0.9300	C38—C39	1.377 (3)
C14—C13	1.385 (4)	C38—C43	1.380 (3)
C15—H15A	0.9700	C39—C40	1.391 (4)
C15—H15B	0.9700	C39—H39A	0.9300
C16—C15	1.509 (3)	C40—H40A	0.9300
C16—C17	1.384 (4)	C41—C40	1.367 (4)
C16—C21	1.376 (3)	C41—C44	1.510 (4)
C17—C18	1.369 (4)	C42—C41	1.369 (4)
C17—H17A	0.9300	C42—H42A	0.9300
C18—H18A	0.9300	C43—C42	1.380 (3)
C19—C18	1.373 (4)	C43—H43A	0.9300
C19—C22	1.511 (4)	C44—H44A	0.9600
C20—C19	1.372 (4)	C44—H44B	0.9600
C20—H20A	0.9300	C44—H44C	0.9600
			
C8—N1—C14	105.0 (2)	C19—C22—H22C	109.5
C8—N2—C15	128.5 (2)	H22A—C22—H22B	109.5
C9—N2—C8	105.8 (2)	H22A—C22—H22C	109.5
C9—N2—C15	125.2 (2)	H22B—C22—H22C	109.5
C30—N3—C36	105.4 (2)	C24—C23—C30	123.7 (2)
C30—N4—C31	106.51 (19)	C28—C23—C24	118.3 (2)
C30—N4—C37	129.1 (2)	C28—C23—C30	118.0 (2)
C31—N4—C37	124.1 (2)	C23—C24—H24A	119.7
C2—C1—C8	121.9 (2)	C25—C24—C23	120.6 (2)
C6—C1—C2	118.5 (2)	C25—C24—H24A	119.7
C6—C1—C8	119.5 (2)	C24—C25—C26	121.3 (2)
C1—C2—C3	120.9 (3)	C24—C25—H25A	119.3
C1—C2—H2A	119.5	C26—C25—H25A	119.3
C3—C2—H2A	119.5	C25—C26—C29	121.4 (3)
C2—C3—C4	120.3 (3)	C27—C26—C25	117.4 (2)
C2—C3—H3B	119.9	C27—C26—C29	121.1 (3)
C4—C3—H3B	119.9	C26—C27—H27A	119.2
C3—C4—C7	120.0 (3)	C28—C27—C26	121.6 (3)
C5—C4—C3	118.6 (3)	C28—C27—H27A	119.2
C5—C4—C7	121.3 (3)	C23—C28—H28A	119.6
C4—C5—C6	121.3 (3)	C27—C28—C23	120.7 (2)
C4—C5—H5A	119.3	C27—C28—H28A	119.6
C6—C5—H5A	119.3	C26—C29—H29A	109.5
C1—C6—H6A	119.8	C26—C29—H29B	109.5
C5—C6—C1	120.4 (3)	C26—C29—H29C	109.5
C5—C6—H6A	119.8	H29A—C29—H29B	109.5
C4—C7—H7A	109.5	H29A—C29—H29C	109.5
C4—C7—H7B	109.5	H29B—C29—H29C	109.5
C4—C7—H7C	109.5	N3—C30—N4	112.4 (2)
H7A—C7—H7B	109.5	N3—C30—C23	121.6 (2)
H7A—C7—H7C	109.5	N4—C30—C23	125.9 (2)
H7B—C7—H7C	109.5	N4—C31—C32	131.9 (2)
N1—C8—N2	113.1 (2)	N4—C31—C36	105.7 (2)
N1—C8—C1	123.4 (2)	C32—C31—C36	122.4 (2)
N2—C8—C1	123.5 (2)	C31—C32—H32A	122.0
N2—C9—C10	131.3 (3)	C33—C32—C31	116.1 (3)
N2—C9—C14	106.1 (2)	C33—C32—H32A	122.0
C10—C9—C14	122.6 (3)	C32—C33—C34	122.2 (3)
C9—C10—H10A	121.7	C32—C33—H33A	118.9
C11—C10—C9	116.6 (3)	C34—C33—H33A	118.9
C11—C10—H10A	121.7	C33—C34—H34A	119.2
C10—C11—C12	121.3 (3)	C35—C34—C33	121.6 (3)
C10—C11—H11A	119.4	C35—C34—H34A	119.2
C12—C11—H11A	119.4	C34—C35—C36	117.4 (3)
C11—C12—H12A	119.3	C34—C35—H35A	121.3
C13—C12—C11	121.4 (3)	C36—C35—H35A	121.3
C13—C12—H12A	119.3	N3—C36—C35	129.7 (2)
C12—C13—C14	118.2 (3)	N3—C36—C31	109.9 (2)
C12—C13—H13A	120.9	C35—C36—C31	120.4 (2)
C14—C13—H13A	120.9	N4—C37—C38	114.20 (19)
N1—C14—C9	110.0 (2)	N4—C37—H37A	108.7
C13—C14—N1	130.1 (3)	N4—C37—H37B	108.7
C13—C14—C9	119.9 (3)	C38—C37—H37A	108.7
N2—C15—C16	115.6 (2)	C38—C37—H37B	108.7
N2—C15—H15A	108.4	H37A—C37—H37B	107.6
N2—C15—H15B	108.4	C39—C38—C43	117.4 (2)
C16—C15—H15A	108.4	C39—C38—C37	119.9 (2)
C16—C15—H15B	108.4	C43—C38—C37	122.6 (2)
H15A—C15—H15B	107.4	C38—C39—C40	120.7 (3)
C17—C16—C15	119.0 (2)	C38—C39—H39A	119.7
C21—C16—C17	117.4 (2)	C40—C39—H39A	119.7
C21—C16—C15	123.7 (2)	C39—C40—H40A	119.2
C16—C17—H17A	119.3	C41—C40—C39	121.6 (3)
C18—C17—C16	121.4 (3)	C41—C40—H40A	119.2
C18—C17—H17A	119.3	C40—C41—C44	121.1 (3)
C17—C18—C19	121.7 (3)	C42—C41—C40	117.6 (3)
C17—C18—H18A	119.2	C42—C41—C44	121.2 (3)
C19—C18—H18A	119.2	C41—C42—C43	121.4 (3)
C18—C19—C20	117.2 (3)	C41—C42—H42A	119.3
C18—C19—C22	120.9 (3)	C43—C42—H42A	119.3
C20—C19—C22	121.8 (3)	C38—C43—H43A	119.4
C19—C20—C21	121.7 (3)	C42—C43—C38	121.3 (2)
C19—C20—H20A	119.2	C42—C43—H43A	119.4
C21—C20—H20A	119.2	C41—C44—H44A	109.5
C16—C21—C20	120.6 (3)	C41—C44—H44B	109.5
C16—C21—H21A	119.7	C41—C44—H44C	109.5
C20—C21—H21A	119.7	H44A—C44—H44B	109.5
C19—C22—H22A	109.5	H44A—C44—H44C	109.5
C19—C22—H22B	109.5	H44B—C44—H44C	109.5

### Hydrogen-bond geometry (Å, °)

**Table:**

*D*—H···*A*	*D*—H	H···*A*	*D*···*A*	*D*—H···*A*
C35—H35A···N3^i^	0.93	2.60	3.491 (3)	162

Symmetry codes: (i) −*x*, −*y*+2, −*z*.
